# A Case Series of Retinal Artery Occlusion: When Time Is of the Essence

**DOI:** 10.7759/cureus.60520

**Published:** 2024-05-17

**Authors:** Mohd Faizal Zokri, Othmaliza Othman

**Affiliations:** 1 Ophthalmology, University Kebangsaan Malaysia Medical Centre, Kuala Lumpur, MYS

**Keywords:** chronic myeloid leukemia (cml), vasospastic syndrome, thromboembolic retina vessel, hypercoagulable state, retinal artery occlusion

## Abstract

This case series discusses the presentation, etiologies, and management of retinal artery occlusions in three patients. The first case was diagnosed as right eye central retinal artery occlusion (CRAO) secondary to a hypercoagulable state as the patient had been newly diagnosed with chronic myeloid leukemia. The second case had right branch retinal artery occlusion (RAO) secondary to a thromboembolic event following a percutaneous transluminal coronary angioplasty procedure. The third case involved a right eye CRAO secondary to vasospastic syndrome. The first case had good visual recovery as the patient presented to us within four hours of the onset. In contrast, the second and third cases presented after seven to eight hours, resulting in poor visual recovery.

Though several measures have been devised to reverse the occlusion, the final visual prognosis still depends on the degree of occlusion and the time of presentation, as late presentation is usually associated with irreversible visual loss. Detection of RAO may require a multidisciplinary team approach, and proper and timely management may reverse the ischemic state of the retina.

## Introduction

The central retinal artery (CRA) is the terminal artery supplying the inner retina [1]. Obstruction of the CRA from an embolus elsewhere in the arterial circulation can result in ischemia and, if left untreated, lead to infarction and permanent visual loss. Central retinal artery occlusion (CRAO) is a well-known ophthalmic and neurologic emergency, involving sudden, painless, and dramatic onset of devastating visual loss with an incidence of 1 per 100,000, as reported from a single county in the US [1].

CRAO is classified into four different clinical entities [2]: (1) Non-arteritic CRAO (NA-CRAO): it involves a classical clinical picture of permanent CRAO with retinal infarction, cherry-red spot, retinal arterial changes, absent or poor residual retinal circulation on fluorescein fundus angiography, and no evidence of giant cell arteritis. This variant accounts for the majority of CRAO cases, seen in about 65% of patients. (2) NA-CRAO with cilioretinal artery sparing: it is associated with a classical clinical picture of permanent NA-CRAO except for the presence of a patent cilioretinal artery in approximately 20% of the population, with a considerable effect on visual prognosis and retinal circulation particularly in the macula regions. (3) Transient NA-CRAO: occlusion of the CRA may vary from several minutes to hours, depending on the causes of obstruction. The visual outcome varies and depends on the duration of transient CRAO. This occurs in around 15% of patients with CRAO, and they carry a 1% annual risk of a sequential NA-CRAO, resulting in irreversible visual impairment [3]. (4) Arteritic CRAO: it shows classical findings of CRAO except for associated optic disc edema following anterior ischemic optic neuropathy secondary to giant cell arteritis leading to the development of permanent CRAO. Less than 5% of CRAO patients exhibit underlying vasculitis, often attributed to giant cell arteritis.

Experimental studies regarding CRAO in old, atherosclerotic, hypertensive rhesus monkeys (similar to the human population with CRAO) have shown that the retina suffers no detectable damage with CRAO for 97 minutes; however, beyond that time frame, the longer the duration of the CRAO, the more extensive the irreversible damage [4]. CRAO lasting for about more than 240 minutes results in massive, irreversible retinal damage [4,5]. As such, the duration of CRAO has emerged as the paramount determinant influencing the development of irreversible retinal damage. We present a series of three cases that will illustrate the clinical course of retinal artery occlusion (RAO) with different etiologies and time of presentation, evaluating how these factors might affect the visual prognosis in these patients.

This case series was presented as a poster at the 38th Asia-Pacific Academy of Ophthalmology Congress (APAO 2023) in Kuala Lumpur, Malaysia.

## Case presentation

Case 1

**Figure 1:**
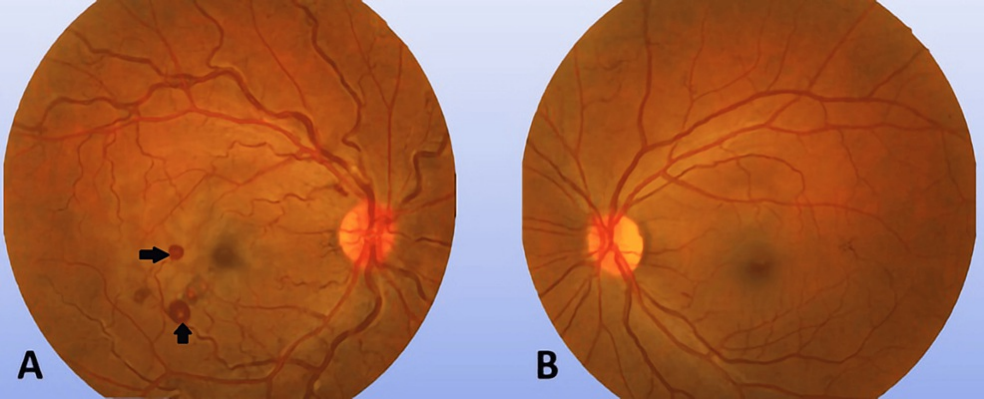
Color fundus photos of both eyes The image showed the right eye to be hyperemic, with blurred nasal disc margin of the optic disc, tortuous and dilated retinal vessels, pale macula, and Roth spots (black arrows) inferotemporal of the macula (A). The left eye fundus was unremarkable (B)

**Figure 2:**
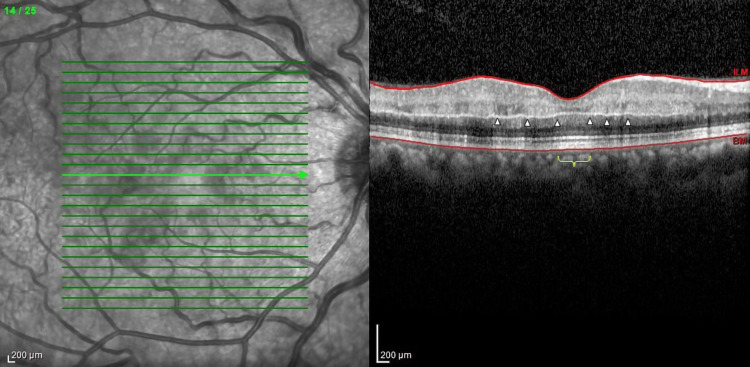
Optical coherence tomography (OCT) of the right eye The image showed hyperreflectivity and thickening of the inner retinal layer. Hyporeflective shadows of the outer retina and choroid in the macula with preserved foveal region (yellow brace) were also noted. Prominent middle limiting membrane (p-MLM) (arrowheads) precisely delineated the areas of ischemic injury

A 41-year-old female, with a known case of newly diagnosed chronic myeloid leukemia, presented with sudden-onset loss of vision in the right eye. Her visual acuity assessment revealed light projection and positive relative afferent pupillary defect (RAPD). Anterior segment findings were normal. The posterior segment revealed a blurred disc margin with tortuous and dilated retinal vessels. The presence of Roth spots at the inferotemporal macula was observed (Figure 1). Anterior and posterior segment examination of her left eye was normal. Optical coherence tomography (OCT) of the right eye showed hyperreflective and diffuse thickening of the inner retinal layers with backscattering of the outer retinal layers (Figure 2). Central macula thickness (CMT) was 283 µm. OCT of her left eye was normal.

The patient had presented to us within four hours of the onset. Her right eye vision improved to 2/60 immediately after performing an ocular massage for around 30 minutes and the initiation of oral acetazolamide. Her vision further improved to 6/60 on the following day. Her white blood cell count was 205.4 x10^9^/L (normal range: 4-10 x 10^9^/L) with neutrophil predominance. The platelet count was 622 x 10^9^/L (normal range: 150 to 410 x 10^9^/L). A full blood analysis showed hyperleukocytosis with granulocytes of all stages of maturation with basophilia and the presence of 2% blast, suggestive of myeloproliferative neoplasm, most likely chronic myeloid leukemia. Her other blood parameters were normal.

The impression was as follows: right eye post reperfusion of CRAO following a hypercoagulable state (chronic myeloid leukemia). She was then co-managed with the hematologist team at a different center due to logistical reasons and was subsequently started on immunosuppressive agents.

Case 2

**Figure 3:**
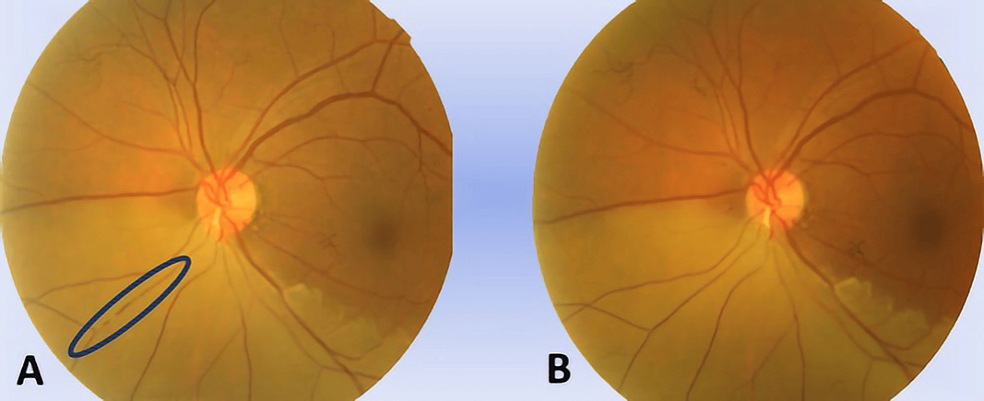
Color fundus photo of the left eye The image showed Hollenhorst plaque at the inferior retinal artery at the optic disc with cattle-trucking at the inferonasal artery (rounded) and pallor over the inferior hemi-retinal area (A). After an ocular massage and oral acetazolamide, cattle-trucking over the inferonasal artery had resolved while pallor over the inferior hemi-retinal area remained (B)

**Figure 4:**
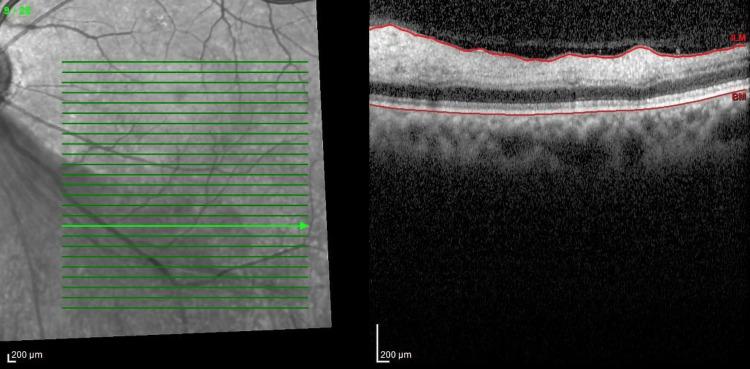
Optical coherence tomography (OCT) of the left eye The image showed hyperreflectivity and thickening of the inner retinal layer at the inferotemporal retina

A 61-year-old male with a known case of hypertension and ischemic heart disease presented with sudden-onset loss of the superior visual field of the left eye during a percutaneous transluminal coronary angioplasty (PTCA) procedure. His left eye vision was 6/36, with positive RAPD, and fundus revealed pale inferior hemi-retinal with attenuated vessels and Hollenhorst plaque at the bifurcation of the inferior retinal artery at the optic disc. There was also cattle-trucking of the inferonasal retinal artery extending nasally (Figure 3). His right eye examination was normal. Left eye OCT showed hyperreflectivity and thickening of the inner retinal layer over inferotemporal areas with loss of organized layered structure of the inner retina (Figure 4). The CMT was 274 µm with preservation of foveal contours.

Our impression was as follows: right eye inferonasal hemi-retinal artery occlusion secondary to dislodged coronary thromboembolic (post-PTCA procedure) with sparing of the cilioretinal artery (superior and inferior). The patient had presented to us eight hours after the onset. Though immediate ocular massage was performed and oral acetazolamide was given, his superior field defect persisted despite vision improving to 6/6 after three days. Upon further review one month later, the superior field defect persisted with a central visual acuity of 6/6.

Case 3

**Figure 5:**
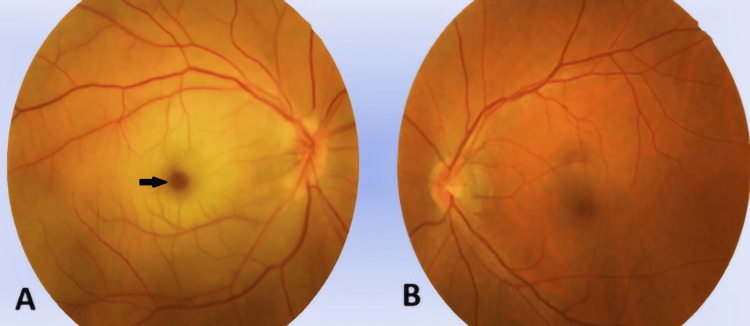
Color fundus photo of both eyes The image showed a blurred disc margin circumferentially, a cherry-red spot at the macula (black arrow), and pallor of the posterior pole; retinal vessels were neither tortuous nor dilated, and no Hollenhorst plaque was seen (A). The left eye fundus was unremarkable (B)

**Figure 6:**
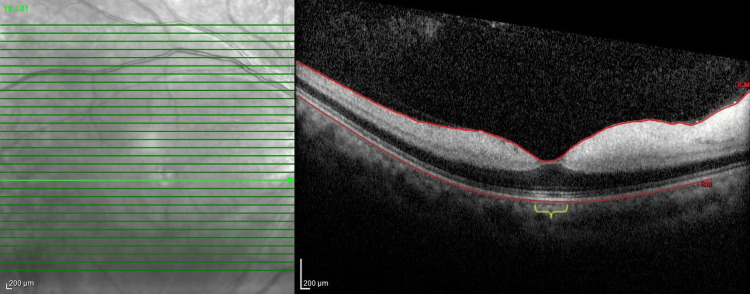
Optical coherence tomography (OCT) of the right eye The image showed hyperreflectivity and thickening of the inner retinal layer nasal to the macula. There was shadowing hyporeflectivity of the outer retina and choroid in the macula area sparing the foveal center (yellow brace)

A 43-year-old female, an endocrine surgeon, with underlying hypertension presented with sudden-onset loss of the right eye vision upon waking up from sleep. She had already experienced two previous episodes of total transient visual loss (two weeks before) that had lasted for a few minutes, which had prompted her to seek ophthalmologist care. Imaging studies at that time had shown no significant findings. She presented to us seven hours after the onset, with the right eye vision analysis showing non-light projection and positive RAPD. The posterior segment revealed a swollen hyperemic disc with a cherry-red spot and a pale posterior pole (Figure 5). OCT showed CMT of 409 µm with retinal thickening and hyperreflectivity of the inner retinal layer suggestive of acute retinal ischemia (Figure 6).

Her vision improved to hand motion after ocular massage and oral calcium channel blocker (CCB) commencement. Coagulation profiles, thrombophilia studies, connective tissue panels, and imaging studies were unremarkable. A diagnosis of right eye CRAO following retinal artery vasospastic syndrome was made.

## Discussion

In the first case, right eye NA-CRAO occurred due to hyperviscosity syndrome secondary to CML. Leukemic infiltration of the optic nerve can be classified into two distinct clinical manifestations [6]: optic nerve head (prelaminar) invasion and optic nerve infiltration behind the lamina cribrosa (retrolaminar). In the former, a fluffy mass of leukemic cells is visible at the optic nerve head and vision typically remains unaffected. The latter clinical manifestation, like in our patient, involves optic disc edema, which usually presents with poor vision and should be differentiated from papilledema secondary to elevated intracranial pressure. The other signs of leukemic infiltration, in this case, were the presence of a few intraretinal hemorrhages with a centered white dot that may represent cellular debris, capillary emboli, or accumulation of leukemic cells [7]. The tortuosity of retinal veins was observed, suggesting the possibility of atypical retinal vein occlusion, probably resulting from a reduction of retinal blood flow caused by NA-CRAO [8].

The OCT of the right eye showed a prominent middle limiting membrane (p-MLM), which is a reliable indicator, especially in cases with indistinct or blotchy inner retinal hyperreflectivity alterations in OCT [9]. The sign of p-MLM in OCT consists of rapid swelling primarily involving the bipolar cell synapses in the inner-outer plexiform layer due to ischemia, which manifests as a hyperreflective band against areas of comparatively mild ischemic retina damage [10]. A prompt referral to a hematologist and targeted therapy for treating the underlying cause of NA-CRAO, which in this case was due to a hypercoagulable state, is crucial to prevent further visual disability.

In the second case, the patient had right eye NA-CRAO with cilioretinal artery sparing secondary to thromboembolic phenomenon following the PTCA procedure. Distal emboli to the heart and kidney have been reported following both coronary and renal artery balloon angioplasty [11]. In a retrospective study of 1500 patients undergoing PTCA [11], 543 (21%) complications were reported, comprising one cerebrovascular event and three transient ischemic attacks. The occurrence of these neurological complications proved that balloon angioplasty may cause embolic events affecting tissues other than the target organ. Two mechanisms may be involved, as follows: (i) the atherosclerotic debris may originate from a pre-existing endothelium vascular lesion resulting directly from catheterization, or (ii) the emboli may result from thrombus formation while exchanging the guidewire [12]. The presence of a cilioretinal artery in this patient proved to be beneficial as his central vision was not affected though the superior field defect remained for a few months, as per his last follow-up. This aligns with a study that described that the transient or cilioretinal artery-sparing NA-CRAO showed relatively spared central visual fields compared to typical NA-CRAO, while central scotoma was the most common type of visual field defect in CRAO [2].

In the third case, the patient had right eye NA-CRAO secondary to vasospastic syndrome likely triggered by presumed emotional stress. This was supported by a negative connective tissue disorder serum panel, and normal imaging findings, and was also attributed to the nature of work, which predisposes her to stress. According to another case report, emotional stress, cold, and exercise have all been shown to precipitate vasospasm-induced blindness [13]. CCB given to this patient has shown efficacy in relieving idiopathic central retinal artery vasospasm [14].

The first case presented earlier (within four hours of onset) whereas the second and third cases presented later (post seven to eight hours), and prompt measures had reversed the ischemic state and improved retinal tissue perfusion in the first case. The duration of CRAO is almost always the principal determining factor behind these patients developing irreversible retinal damage.

The use of OCT in detecting ischemic features is indispensable for validating ischemic damage areas in the initial stage of RAO, as this helps detect the thickening of the inner retinal layers in acute ischemic regions. However, increased reflectivity has been reportedly the more obvious feature [15,16]. As time goes by, the previously observed inner retinal layer hyperreflectivity and thickening in OCT, caused by ischemic swelling, subside, and are replaced by retinal atrophy. The reproducibility of OCT findings and the fact that it is readily available and time-saving make it an efficient tool for monitoring the improvement and visual prognosis of RAO.

## Conclusions

This case report highlighted the importance of providing continuous education to eyecare providers and emergency medical services and raising awareness among patients regarding the acute presentation of CRAO, so that prompt emergency treatment based on the underlying etiologies is ensured. The timing of presentation is very important as early detection will result in better outcomes in terms of both visual acuity and improvement of visual field defect. A multidisciplinary approach is necessary to find the underlying etiology, especially given the high mortality rates associated with this condition. Despite the grim prognosis for CRAO, efforts to re­store vision, regardless of the therapy used, should be made, preferably within four hours of symptom onset. This may subsequently improve retinal perfusion and prevent permanent visual disabilities, and time is really of the essence in managing these patients.
